# Oleuropein Reverses Repeated Corticosterone-Induced Depressive-Like Behavior in mice: Evidence of Modulating Effect on Biogenic Amines

**DOI:** 10.1038/s41598-020-60026-1

**Published:** 2020-02-24

**Authors:** Amira M. Badr, Hala A. Attia, Nouf Al-Rasheed

**Affiliations:** 1 Department of Pharmacology and Toxicology, College of Pharmacy, King Saud University, Riyadh, Saudi Arabia; 2 Department of Pharmacology and Toxicology, College of Pharmacy, Ain Shams University, Heliopolis, Cairo, Egypt; 3 Department of Biochemistry, Faculty of Pharmacy, Mansoura University, Mansoura, Egypt

**Keywords:** Depression, Neurology

## Abstract

Depression is still one of challenging, and widely encountered disorders with complex etiology. The role of healthy diet and olive oil in ameliorating depression has been claimed. This study was designed to explore the effects of oleuropein; the main constituent of olive oil; on depression-like behaviors that are induced by repeated administration of corticosterone (40 mg/kg, i.p.), once a day for 21 days, in mice. Oleuropein (8, 16, and 32 mg/kg, i.p.) or fluoxetine (20 mg/kg, positive control, i.p.1) was administered 30 minutes prior to corticosterone injection. Sucrose consumption test, open-field test (OFT), tail suspension test (TST), and forced swimming test (FST) were performed. Reduced Glutathione (GSH), lipid peroxidation, and biogenic amines; serotonin, dopamine, and nor-epinephrine; levels were also analyzed in brain homogenates. Corticosterone treatment induced depression-like behaviors, it increased immobility time in the TST, OFT, and FST, decreased the number of movements in OFT, and decreased sucrose consumption. Corticosterone effect was associated with depletion of reduced glutathione and increase of lipid peroxidation, in addition to modification of biogenic amines; decreased serotonin and dopamine. Oleuropein or fluoxetine administration counteracted corticosterone-induced changes. In conclusion, oleuropein showed a promising antidepressant activity, that is evident by improving corticosterone-induced depression-like behaviors, and normalizing levels of biogenic amines.

## Introduction

Major depressive disorder (MDD), also known as depression, is a common mental disorder that affects patients’ health and quality of life, being associated with psychological, social and physical problems, as well as suicidal tendency^1^. It has a complex biological pattern of etiology, involving genetic and epigenetic factors, in addition to various environmental stressors^2^. Recent evidences suggest that oxidative stress might contribute significantly to the pathogenesis of many psychiatric disorders, including depression. This was supported by data reporting that major depression is associated with lowered levels of several endogenous antioxidants, including vitamin E, zinc and coenzyme Q10, along with reduced antioxidant enzymes such as glutathione peroxidase^3^. Moreover, reactive oxygen species (ROS) and reactive nitrogen species (RNS) have been shown to modulate the levels and activities of biogenic amines; norepinephrine, serotonin, and dopamine^4^. As those biogenic amines represent the principal neurotransmitters implicated in the pathogenesis of depression, so these findings support that antioxidants may play a role as alternative therapeutic modalities and represent new potential targets for treatment of depression^5^.

Clinical trials showed that Mediterranean diet is correlated with low incidence of depression, and high level of brain derived neurotrophic factor. One of the widely used oils in Mediterranean diet is olive oil, which has been suggested to be the major contributor to the improvement observed in depressive symptoms^6,7^. Oleuropein is considered the most active phenolic active ingredient in olive oil. The pharmacological activity of oleuropein is variable, including anti-inflammatory, anti-atherosclerotic, anti-cancer, antiviral and antimicrobial activity^8,9^. Oleuropein has strong, dose-dependent antioxidant activity, it has the ability to scavenge nitric oxide, decrease levels of ROS and RNS, and reduce lipid peroxidation level in various models of ischemia^10^. Antioxidant activity of oleuropein may be explained in terms of its ability to chelate metal ions, inhibit inflammatory enzymes, such as lipoxygenases, and reduce inflammatory mediators, such as tumor necrosis factor-α, nuclear factor-kB, and interleukin 1β (IL-1β) and IL-6^11,12^.

Oleuropein showed a promising neuroprotective effect in different diseases. In Parkinsonism, injection of oleuropein for 6 months in aged rats increased the number of neurons in substantia nigra. It has also showed protective effect in Alzheimer’s disease, and reduced β-amyloid formation^13^. Moreover, a study using olive leaf extract revealed an increase in brain derived neurotrophic factors; which are proteins involved in neurogenesis^14^. Oleuropein is about 50–60% absorbed in humans, and it was shown that the hydroxytyrosol; an active metabolite of oleuropein; is found in the brain of mice following oleuropein oral administration. This approves the ability of oleuropein and/or its active derivatives to cross the blood brain barrier, and support that oleuropein can be used orally as a neuroprotective agent^9,15^. Therefore, the aim of the current study is to examine the anti-depressant effects of oleuropein in a corticosterone-model of depression and explore oleuropein effect on brain-biogenic amines level.

Due to the complexity of MDD in humans, the development of animal models has been difficult so far. Corticosterone (Cort)-induced depression model in rodents has been recently developed, and approved to be a useful and reliable one^2,16^. A large number of evidences showed that human stress experience contributes to the pathogenesis of depression, and may play a role in its degree and potential of recurrence. It was found that depressed patients experience overactive hypothalamic–pituitary–adrenal (HPA) axis, with increased cortisol level^17^. In experimental animals, repeated Cort injection induced depressive-like behaviour, as evidenced by a reduced sucrose consumption, and increased immobility time in behavioral tests, e.g. forced swimming test and tail suspension test. It also induces neurochemical and histopathological changes, that are indicative of depression^3,18^, and are significantly ameliorated by antidepressants^2,18^. Therefore, Cort-induced model of depression is a reliable model for exploring and studying potential antidepressants, and was used in this study. Depressive disorder has been strongly linked to abnormalities in brain 5-hydroxy tryptamine (5-HT or serotonin) activity, and selective serotonin reuptake inhibitors (SSRIs) represent one of the well-known antidepressants^19,20^. Fluoxetine, one of classical SSRIs, was used in this study as a positive control.

## Materials and Methods

### Animals

Male mice (20–25 g) from the Animal Care Center, King Saud University, Riyadh, Saudi Arabia, were housed in standard polypropylene cages (8/cage), and acclimatized for 1 week before starting the experiments. Animals were allowed free access to water and standard basal diet ad libitum and were kept under controlled conditions of temperature (24 ± 1 °C), humidity and 12:12 h light/dark cycle. Handling of animals was in compliance with the guidelines for the care and use of animals for scientific purposes. The study was approved by the Research Ethics Committee at King Saud University, Ethics Reference No. KSU-SE-19-34.

### Drugs and drug administration

Oleuropein was obtained from Extrasynthese® (Genay, France). Fluoxetine, 5-HT, noradrenaline, and dopamine were purchased from Sigma Chemical Co.® (St. Louis, MO, USA). Cort injectable formulation was obtained from local pharmacy in Riyadh (Solu-Cortef®; Pfizer, USA). Other chemicals were of high analytical grade and purchased from Sigma-Aldrich Co. (St. Louis, MO, USA). Oleuropein and fluoxetine were dissolved in normal saline. Cort injectable solution was also diluted with normal saline for dosage adjustment.

### Experimental design

Mice were randomly divided into six groups of eight animals each as follows: Group 1: Mice were given normal saline (i.p.) and considered as the vehicle control. Group 2 (Model group): Mice received Cort (40 mg/kg, i.p.), and normal saline daily for 21 days^18^. Group 3 (Positive control): Mice were injected with Cort (40 mg/kg, i.p.) and fluoxetine (20 mg/kg, i.p.) daily for 21 days^16,18–20^. Groups 4 to 6: Mice in these groups were injected with Cort (40 mg/kg, i.p.) and treated with oleuropein (8, 16, and 32 mg/kg, i.p.), respectively daily for 21 days^21^. Fluoxetine or oleuropein was given 30 min prior to cort injection.

### Behavioral assessments

#### Tail suspension test

The tail suspension test (TST) was carried out according to the method of Steru *et al*.^22^. Animals were suspended 50 cm above the table by an adhesive tape that was placed approximately 1 cm from the tip of the tail. The time the mice remained immobile was recorded during a test period of 6 min. Mice were considered immobile when they hang passively and completely motionless.

#### Forced swimming test

The forced swimming test (FST) used was similar to that described by Porsolt *et al*. (197, 1978) with slight modification^23,24^. This test was performed to assess the despair behaviour of the mice. A pretest was conducted on the 20th day of the experiment, mice were forced to swim for 15 min, in transparent glass cylinders (height: 25 cm; diameter: 10 cm; containing 10 cm of water at 24 ± 1 °C, at this depth, mice could not touch the bottom of the cylinders with either tails or hind limbs.). The mice were then removed, dried and returned back to cages. Twenty-four hour later, the mice were returned to the cylinders for 5 min; test session; and the immobility time was quantified for each mouse by two inspectors blind to the treatment given to each mouse. The session was recorded using a video camera to tape behavior for later manual scoring confirmation. A mouse was considered immobile when remained floating motionless in the water, with only occasional alternate movements of paws and tails needed to keep its head above water.

#### Open field test (OFT)

Ambulatory behavior was assessed in an OFT^25^. The apparatus is a large open field (40 cm × 40 cm × 30 cm) divided into 16 equal squares drawn on the floor of the arena. The test was performed one hour after the last dose of Cort, and before FST- test session. A single mouse was placed in the center of the floor, and the number of squares crossed (with the four paws), and immobility time were recorded for 5 minutes.

#### Sucrose preference test

The sucrose preference test (SPT) was performed to evaluate anhedonia (the inability to feel pleasure), a characteristic symptom of major depression. The SPT was carried out on the 19th day. The test was performed as described previously by Wang *et al*., 2008, with slight modifications^26^. First, the mice were allowed to adapt to sucrose solution (1%, w/v) by putting two bottles of sucrose solution in each cage for 24 h. Second, the mice were housed individually, and one of the bottles was replaced with water, and each bottle contained a specific volume = 100 ml. Sixty minutes later, the volume (ml) of the consumed sucrose solution and that of water were measured, and sucrose preference was calculated as the percentage of sucrose consumption out of total liquid consumption:

$$\begin{array}{ccc}\text{Sucrose preference}\,({\rm{ \% }}) & = & (\text{sucrose consumption}\,({\rm{m}}{\rm{l}})/[\text{sucrose consumption}\,({\rm{m}}{\rm{l}})\\  &  & +\,\text{water consumption}\,({\rm{m}}{\rm{l}})])\times 100.\end{array}$$

### Collection of brain samples

Animals, in different groups, were sacrificed by decapitation on day 21 after carrying out the behavioural tests. The brain tissues were rapidly removed and frozen on dry ice. The tissue samples were weighed and stored at −80 °C until homogenization. For homogenization, brain samples were homogenized in 0.9% normal saline to prepare 20% homogenate and then centrifuged (4οC) at 3000 rpm for 10 min. The supernatants were aliquoted and stored at −80 °C for the assays of reduced glutathione (GSH), lipid peroxides (LPO), and monoamines.

#### Determination of reduced glutathione and lipid peroxides

Lipid peroxides (LPOs) were determined spectrophotometrically as thiobarbituric acid-reactive substances (TBARs), according to the method described by Mihara and Uchiyama^27^. The determination of TBARs was based on the reaction of malondialdehyde with thiobarbituric acid at low pH and high temperature. The pink product was then extracted with n-butanol, and the colorimetric determination was carried out at 535 nm.

Brain homogenate was also used for the determination of GSH as a natural non-enzymatic antioxidant. The assay procedure involved the estimation of GSH using a spectrophotometer according to Ellman’s method^28^. Aliquots of 0.5 ml of tissue homogenates were used. Proteins were precipitated using trichloroacetic acid, and samples were centrifuged at 3000 rpm for 15 min. The supernatant was used for spectrophotometric determination of GSH using Ellman’s reagent at 412 nm.

#### Determination of monoamines

The levels of 5-HT, noradrenaline, and dopamine were measured as described previously^29^, using high-performance liquid chromatography (HPLC) with electrochemical detection with minor modifications. Each frozen tissue sample was homogenized by ultrasonication in 200 μl of 0.4 M perchloric acid (solution A). The homogenate was kept on ice for 1 h and then centrifuged at 11,000 × g (4 °C) for 20 min. The pellet was discarded. An aliquot of 160 μl of supernatant was added to 80 μl of solution B (containing 0.2 M potassium citrate, 0.3 M dipotassium hydrogen phosphate and 0.2 M EDTA). The mixture was kept on ice for 1 h and then centrifuged at 11,000 × g (4 °C) for 20 min again. Twenty μl of the resultant supernatant was directly injected into an ESA liquid chromatography system equipped with a reversed-phase C18 column (150 × 4.6 mm I.D., 5 μm) and an electrochemical detector (ESA CoulArray, Chelmstord, MA, USA.). The detector potential was set at 50, 100, 200, 300, 400, 500 mV, respectively. The mobile phase consisted of 125 mM citric acid–sodium citrate (pH 4.3), 0.1 mM EDTA, 1.2 mM sodium octanesulfonate and 16% methanol. The flow rate was 1.0 ml/min. The tissue levels of monoamine were expressed in terms of nanograms per gram of tissue.

### Statistical analysis

Data were expressed as the mean ± SEM. Significant differences between means were analyzed by one-way analysis of variance (ANOVA) and Student’s t-test using GraphPad Prism version 5 (GraphPad Software). P-value ≤ 0.05 was considered significant.

## Results

### Behavioral tests

#### Effect of oleuropein on immobility time in TST

**Figure 1:**
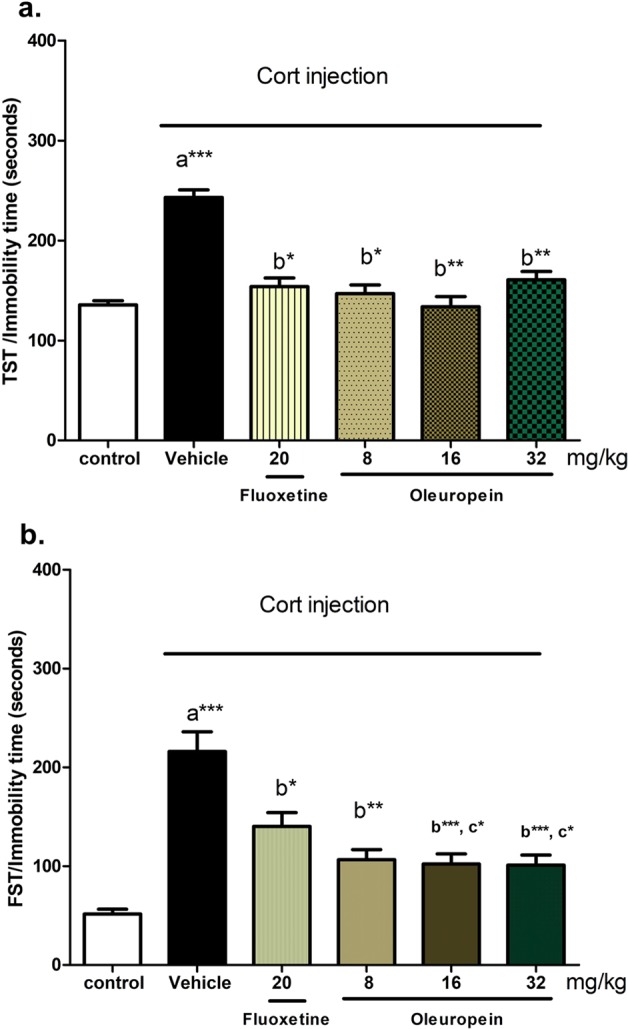
Effects of different doses of oleuropein on immobility time in chronic Cort-induced depression model. (**a**) Tail Suspension Test (TST) and (**b**) Forced Swimming Test (FST) in different animal groups. Each bar represents the mean of 6 rats + SEM. a: significant compared to the control group; b: significant compared to the Cort group; c: significant compared to Cort + Fluoxetine group; d: significant compared to Cort + oleuropein (8 mg/kg) group. *p ≤ 0.05, **p ≤ 0.01, ***p ≤ 0.001.

Treatment with Cort caused a significant increase in the immobility time of rats in the TST (80%, p < 0.001) compared with the control group (Fig. 1a). Fluoxetine (20 mg/kg), and oleuropein treatment at 8, 16 and 32 mg/kg significantly decreased the immobility time compared with the Cort-treated mice (Fig. 1a), and the levels were comparable to the control group. No significant differences were observed between different doses of oleuropein, as well as between oleuropein (at different doses) and fluoxetine.

#### Effect of oleuropein on immobility time in FST

Cort injection increased immobility time significantly in FST compared to that of normal control (315%, p < 0.001), Fig. 1b. The immobility time during 5 minutes were significantly reduced by the long-term treatment for 21 days) with fluoxetine (p < 0.05) and, to greater extent, with oleuropein at 8 mg/kg (p < 0.01), 16 mg/kg (p < 0.001) and 32 mg/kg (p < 0.001) compared to Cort-treated mice. Oleuropein treatment at doses of 16 and 32 mg/kg caused a significant improvement comparable to that of fluoxetine treatment (p < 0.05). Oleuropein at 16 and 32 mg/kg caused about 50% reduction in immobility time compared with that of Cort group (p < 0.001).

#### Effect of oleuropein on immobility time and number of movements in OFT

**Figure 2:**
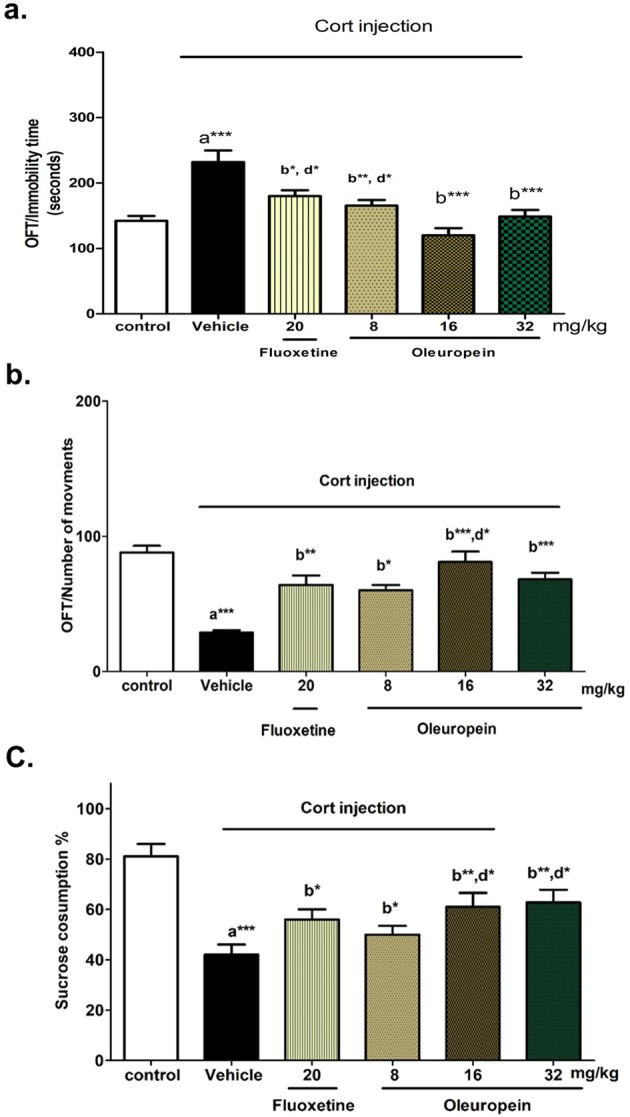
Effects of different doses of oleuropein on chronic Cort-induced depression model. (**a**) Immobility Time in Open Field Test (OFT), (**b**) Number of Movements in Open Field Test (OFT), and (**c**) Sucrose Consumption % in Sucrose Preference Test in different animal groups. Each bar represents the mean of 6 rats + SEM. a: significant compared to the control group; b: significant compared to the Cort group; c: significant compared to Cort + Fluoxetine group; d: significant compared to Cort + oleuropein (8 mg/kg) group. *p ≤ 0.05, **p ≤ 0.01, ***p ≤ 0.001.

Immobility Time. Cort treatment significantly increased immobility time in OFT. It caused about 65% increase (p < 0.001) compared to control group (Fig. 2a). This increase was significantly attenuated by treatment with fluoxetine (p < 0.05) and, to a higher extent, with oleuropein at 8 mg/kg (p < 0.01), 16 mg/kg (p < 0.001) and 32 mg/kg (p < 0.001). In addition, oleuropein at doses 16, and 32 mg/kg reduced immobility time significantly compared to fluoxetine (p < 0.05), and oleuropein dose of 16 mg/kg showed a significant difference from that of 8 mg/kg (p < 0.05).

Number of Movements. In accordance with previous results, Cort treated mice showed decreased number of movements by about 68% (p < 0.001) as compared to normal control. Pretreatment with either Fluoxetine, or oleuropein at different doses increased number of movements significantly as compared to Cort treated mice. Oleuropein at doses 16 and 32 mg/kg maintained number of movements comparable to the untreated control group (Fig. 2b).

#### Effect of oleuropein on anhedonic behavior in SPT

In the present study, sucrose preference in mice was calculated. The repeated administration of Cort significantly reduced sucrose preference as compared to control-untreated group (Fig. 2c). However, mice treated with oleuropein at different doses exhibited a significant inhibition of the decrease in consumed sucrose intake as compared to the Cort group, with doses 16 and 32 mg/kg shows significant inhibition compared to the dose of 8 mg/kg (p <  0.01, 50% inhibition of the decrease in the consumed sucrose). Fluoxetine, also, increased the sucrose preference in the rat compared to Cort (p <  0.05, 42% increase in sucrose consumption).

### Biochemical measurements

#### Effect of oleuropein on reduced glutathione and lipid peroxides

**Figure 3:**
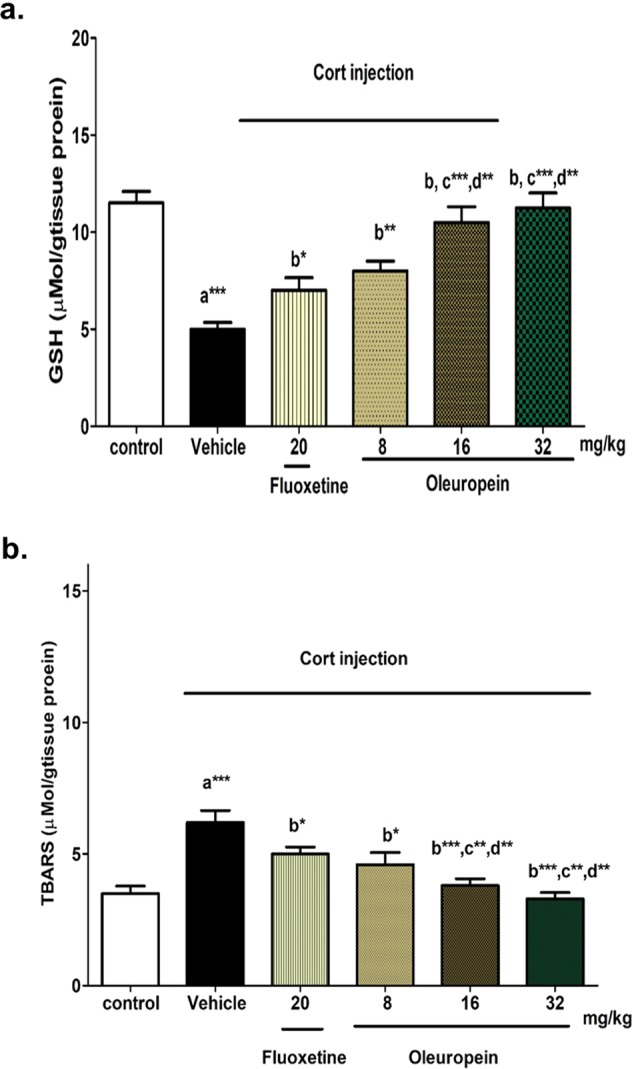
Effects of different doses of oleuropein on oxidative stress-related parameters in chronic Cort-induced depression model. (**a**) Reduced Glutathione (GSH); u Mol/g tissue protein; and (**b**) Thiobarbituric Acid Reactive Substances (TBARS); u Mol/g tissue protein in different animal groups. Each bar represents the mean of 6 rats + SEM. a: significant compared to the control group; b: significant compared to the Cort group; c: significant compared to Cort + Fluoxetine group; d: significant compared to Cort + oleuropein (8 mg/kg) group. *p ≤ 0.05, **p ≤ 0.01, ***p ≤ 0.001.

Cort treatment induced a significant decrease in GSH, with a significant increase in TBARS (the marker of lipid peroxidation) compared to the normal control group (65% decrease, and 2 fold increase, p < 0.001, respectively) reflecting oxidant/antioxidant imbalance. The decreased GSH and elevated TBARS were attenuated by the pretreatment with oleuropein, or fluoxetine compared to Cort-treated mice. The higher doses of oleuropein (16 and 32 mg/kg) revealed significant improvement compared to the lowest dose, 8 mg/kg (p < 0.001), and was comparable to that of the control. Moreover, oleuropein at doses 16 and 32 mg/kg showed significant higher brain levels of GSH, and lower TBARS level compared to that of fluoxetine, the positive control group (Fig. 3).

#### Effect of oleuropein on brain levels of monoamines

**Figure 4:**
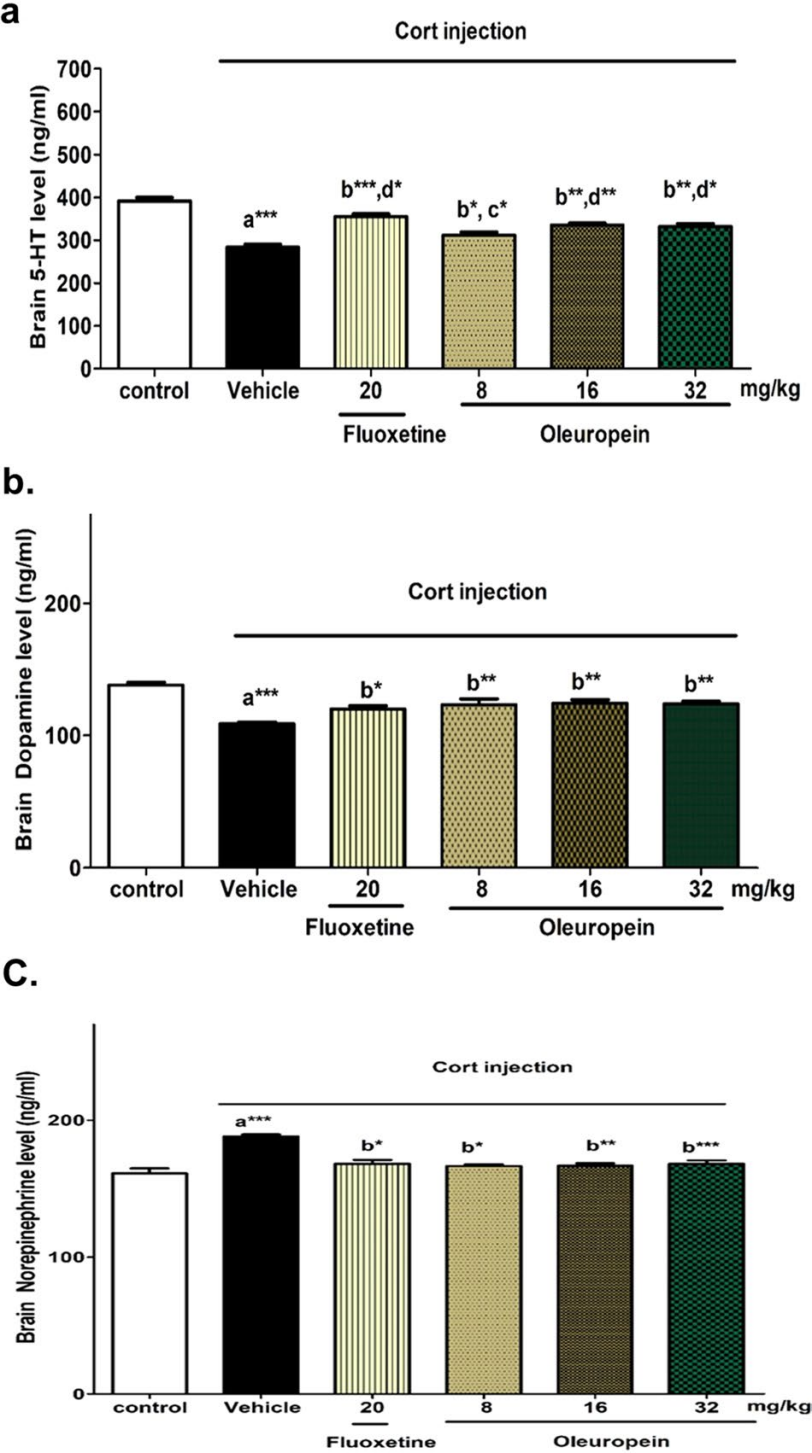
Effects of different doses of oleuropein on brain neurotransmitters’ level in chronic Cort-induced depression model in mice. (**a**) Brain Serotonin (5-HT) Level (ng/ml), (**b**) Brain Dopamine Level (ng/ml), and (**c**) Brain Norepinephrine Level (ng/ml) in different animal groups. Each bar represents the mean of 6 rats + SEM. a: significant compared to the control group; b: significant compared to the Cort group; c: significant compared to Cort + Fluoxetine group; d: significant compared to Cort + oleuropein (8 mg/kg) group. *p ≤ 0.05, **p ≤ 0.01, ***p ≤ 0.001.

Mice treated with Cort for 21 days showed significantly reduced levels of serotonin and dopamine as compared to that of the control group (approximately 27% reduction, p < 0.001) (Fig. 4a,b, respectively). Pretreatment with either oleuropein or fluoxetine ameliorated Cort-induced decrease of serotonin and dopamine. The pretreatment with fluoxetine and higher doses of oleuropein (16 and 32 mg/kg) significantly increased the brain levels of serotonin compared to the low dose of oleuropein (8 mg/kg).

On the contrary, Cort-injected mice exhibited a significantly elevated brain level of norepinephrine compared to the normal control (about 15% increase, p < 0.001). However, mice pretreated with oleuropein and fluoxetine exhibited lowered norepinephrine level significantly as compared to Cort- treated mice (Fig. 4c). No significant differences in norepinephrine levels were observed among different pretreated mice.

## Discussion

MDD is a disabling mental illness affecting increasingly large population, and is considered the highest prevalence psychiatric disorder, with a relatively high morbidity^30^. Oxidative stress has been reported in humans and animals under stressful stimuli^4^. An increasing body of evidence points to the role of oxidative stress in changing the neuronal plasticity that accompanies MDD, and thus the potential role of antioxidants in the management of depression^31^. Recent evidences confirmed that some antidepressants, like fluoxetine and mirtazapine, possess antioxidant activities^5,20,31–33^. In accordance, many strong well-known antioxidants showed to have antidepressant activity, e.g. Quercetin and Vitamin E^34,35^. Therefore, the aim of the present study was to investigate the effect of different doses of oleuropein, a strong antioxidant derived from olives, in ameliorating depressive-like behaviors in a Cort-model of depression, and to explore its effect on biogenic amines. Cort model of depression closely displays many of the characteristic depressive symptoms and has shown to be a reliable model for identifying antidepressants, in addition to studying the implicated pharmacological mechanisms^36^. In our knowledge, our study is the first to identify the effect of different doses of oleuropein on depression-like behaviors and biogenic amines on a depression-induced animal model.

In the current study, FST and TST were used to evaluate the depressive-like behaviors, and OFT for anxiety-like behaviors. FST is considered one of the most commonly used antidepressant evaluation tests, with high predictivity and sufficient reliability. It also discriminates antidepressants from neuroleptics^37^. In this regard, the 21-day schedule of daily Cort injection significantly induced depression-like behaviors, as revealed by the significantly increased immobility time in both TST and FST (Fig. 1), which was in accordance with previous studies^38,39^. In addition, Cort treatment induced an anxiety-like behavior as detected by decreasing the number of movements and increasing the immobility time in OFT (Fig. 2a,b), that was in harmony with the results of Sturm *et al*.^40^, and Wei *et al*.^41^. Treatment with oleuropein at different doses reversed the effect of Cort on TST, FST, and OFT, with the best improvement with the doses 16 and 32 mg/kg, and particularly with the dose 16 mg/kg that showed significant differences in OFT test compared to the dose 8 mg/kg. This data demonstrates the beneficial effects of oleuropein on depressive and anxiety-like behavior-induced by stressors resembled by Cort injection. Previously, olive oil was found to induce a significant reduction of immobility time in FST in reserpine-induced depression in male rats^42^. According to our results, this effect of olive oil may be attributed to its oleuropein content.

In accordance with previous studies, fluoxetine treatment reversed the behavioural effects of Cort treatment in the TST, FST and OFT^19,39^. Interestingly, the effects of oleuropein 16 mg/kg was comparable, and even superior regarding FST, to that of classical antidepressant drug; fluoxetine; supporting that 16 mg/kg is the most effective dose. The variation in the immobility time after chronic oleuropein treatment may reflect changes in HPA axis function.

The SPT is being considered as a simple test evaluating anhedonia behavioural change, which is defined as a characteristic symptom of MDD in humans^43^. It is detected by diminished response to rewards, such as the reduced consumption of naturally preferred sweetened beverages^44^. In consistent with previous reports^38,45^, chronic Cort injection in the current study significantly decreased the sucrose preference, while oleuropein at different doses attenuated this decrease with the best improvement with the doses of 16 and 32 mg/kg and their effects were comparable to that of fluoxetine (Fig. 2c). The effect of fluoxetine on SPT was in accordance with other studies^46,47^. Our results clearly demonstrate the potential positive effects of oleuropein on depressive-like behaviours, anxiety and anhedonia.

To explore the possible mechanisms of action of oleuropein, the effects of oleuropein treatment on one of the essential endogenous antioxidants, GSH, and an oxidative stress indicator, TBARS, were assayed. Increased free radicals production can eventually lead to cell death and consequently atrophy of vulnerable neurons in hippocampal and frontal cortex^48^. Therefore, a strong correlation between increased oxidative stress and depressive disorder has been postulated and supported by the increased antidepressant activity of several antioxidants in different models of depression^49,50^. In addition, brain cells contain a very high content of polyunsaturated fatty acid (PUFA), which together with the high energy requirements and oxygen consumption, make the brain highly susceptible to oxidative stress^51^. In the current study, Cort-induced oxidative stress was evident by a significant increase of TBARS (an index of PUFA oxidation), which was associated with GSH depletion (Fig. 3). The effect of Cort injection on lipid peroxidation and GSH is in agreement with that reported before by Chen^52^, and Zeni^53^.

The antioxidant activity of oleuropein was partially demonstrated by the significant amelioration of elevated TBARS, and correction of depleted GSH compared to Cort group. The doses of 16 and 32 mg/kg offered greater effects compared to that of 8 mg/kg. As expected, the effect of oleuropein, on GSH and TBARS, was superior to that of fluoxetine. Fluoxetine ameliorated Cort-induced lipid peroxidation and GSH depletion, and this was in accordance with Jayakumar *et al*., and Rebai *et al*.^54,55^. The antioxidant activity of oleuropein was previously reported in various tissues^56,57^ and in the brain^58,59^. Oleuropein is a potent scavenger of peroxynitrite and superoxide anion radicals, as well as hydroxyl radicals, and its peroxyl radicals scavenging activity was twice that of Trolox^60,61^.

The three main brain monoamines (noradrenaline, 5-HT and dopamine) regulate several behavioral functions and play critical role in learning, sleep, mood, anxiety, motor coordination, and learning^54^. An association between their deficiency and the pathogenesis of depression and its symptoms has been suggested^62^. In the current study, dopamine and serotonin levels were reduced after chronic exposure to Cort. Our results are supported by previous data reporting that prolonged exposure to high Cort. levels diminishes dopamine and serotonin responses in the hippocampus^63,64^. In addition, Ago *et al*., 2008 found that antagonism of glucocorticoid receptor is associated with antidepressant-like effects, via interfering with dopaminergic neurotransmission in Cort-treated mice^65^, suggesting that the dopaminergic neurotransmission contributes to the depressive-like behaviors induced in Cort-treated mice.

It has been reported that drugs that elevate the levels of monoamines elicit antidepressant effects (according to the monoamine theory of depression)^66^, and many available antidepressants act via increasing biogenic amines especially serotonin, based on biogenic amine theory of depression. Therefore, the effect of oleuropein on the biogenic amines was evaluated. In our study, treatment with either oleuropein or fluoxetine increased dopamine and serotonin to control comparable levels. The effect of oleuropein was parallel to that of SSRI; fluoxetine, which could explain and support the antidepressant activity of oleuropein via modulating dopamine and serotonin reuptake. The mimicking effect of oleuropein on monoamine levels in our model may explain the previous role of olive oil in enhancing the brain levels of norepinephrine, serotonin, and dopamine in reserpine-induced depression in rats^42^.

Un-expectantly, Cort induced a significant increase in cerebral norepinephrine level. This finding was supported by Fan *et al*., 2014 who reported that Cort treatment for 21 days induced a significant increase in dopamine-β-hydroxylase protein (DβH) and nor-epinephrine transporter protein expression (NET) in the brain, and that this effect was counteracted by concomitant treatment with a corticosteroid receptor antagonist (mifepristone), in addition to spironolactone^67^. The changes of NET and DβH expression that was induced by Cort is a normal response to stress and was accompanied by increased stressful behaviours^68^. The effect of Cort on norepinephrine was minimized by treatment with oleuropein or fluoxetine and maintained nor-epinephrine level compared to that of the control group. This may be explained by stress-minimizing activity of oleuropein.

## Conclusions

The current study examined the potential antidepressant-like effects of oleuropein against Cort-induced depressive-like behaviors in male mice. Our findings revealed that oleuropein can be considered as a promising natural compound to ameliorate depressive-like behaviors. The antidepressant-like effects of oleuropein treatment can be explained in terms of maintaining levels of cerebral biogenic amines and reduced glutathione, and inhibiting lipid peroxidation. By comparing the effect of oleuropein treatment to that of fluoxetine, we found that the effects of oleuropein is comparable and even better in some parameters than that of fluoxetine. Further studies are needed to explore the mechanism of action of oleuropein on different pathways involved in depression, such as BDNF and inflammatory pathway.

### Study limitations

One of the major limitations in our study is the assay of biogenic amines in the homogenate of whole brain. Our next plan is to detect in which brain area the oleuropein elicit its beneficial effect on monoamines. Since, antidepressants targeting monoamines directly affect the functional tone of these circuits, notably in limbic and frontal cortical areas, therefore, the concentration of monoamines in these areas will be measured in our ongoing research.

## Data Availability

Data and protocols are available for readers.
